# Exploring the potential of a thermosensitive *in situ* gel with *Periplaneta americana* extracts for efficient wound healing

**DOI:** 10.3389/fphar.2025.1672818

**Published:** 2025-09-01

**Authors:** Shuai Liu, Shuohan Xu, Hang Lu, Xueyu Gong, Huaipeng Li, Xinyu Fan, Hongwei Yang

**Affiliations:** ^1^ Organ Transplant Center, General Hospital of Northern Theater Command, Shenyang, China; ^2^ Department of Radiation Oncology, General Hospital of Northern Theater Command, Shenyang, China; ^3^ Department of Emergency Medicine, General Hospital of Northern Theater Command, Shenyang, Liaoning, China; ^4^ Department of Pharmacy, Shengjing Hospital of China Medical University, Shenyang, China

**Keywords:** *Periplaneta americana* extract, thermosensitive, *in situ* gel, wound healing, MAPK

## Abstract

The primary aim of this study was to fabricate a wound healing thermosensitive *in situ* gel containing *Periplaneta americana* extract (PAE), which is widely utilized clinically due to its anti-inflammatory and skin regeneration effects. The thermosensitive *in situ* gel was prepared using F127 and F68 at concentrations of 17.5% (w/v) and 3.75% (w/v) respectively. The physicochemical properties of the PAE-loaded gel was characterized by DSC, scanning electron microscopy, and FTIR. Additionally, the *in vitro* release behavior and the *in vivo* wound healing effects were also investigated. It was observed that the thermosensitive *in situ* gel exhibited a sol-gel transition temperature of approximately 32.5 °C ± 0.4 °C, and the PAE existed in an amorphous state within the lyophilized gel. FTIR spectra indicated the formation of hydrogel bonds between PAE and F127/F68, signifying a sustained release profile. Further *in vivo* wound healing and inflammatory cytokine experiments revealed that the drug-loaded hydrogel demonstrated superior wound healing activity compared to other counterparts, by accelerating the reduction of endogenous inflammatory mediators such as TNF-α, MMP9, and IL-6, and inhibiting the MAPK/NF-κB pathway. The PAE-loaded thermosensitive *in situ* gel represents a promising pharmaceutical candidate for local wound healing applications, owing to its prolonged retention time.

## Graphical Abstract

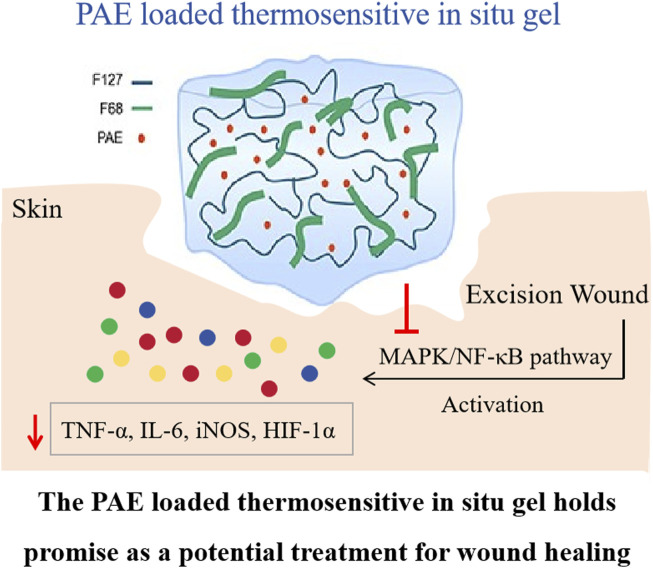

## 1 Introduction

Acute wounds, including scalds, skin burns, traumatic, or surgical injuries, typically undergo a complex cascade of overlapping events such as inflammatory reaction, tissue regeneration, and tissue repair (Sag et al., 2018). Generally, therapeutic strategies are focused on wound coverage to maintain closure, prevent infection, and alleviate pain (Priya et al., 2016). Despite the development of numerous natural or synthetic ointment-based wound dressing formulations, there are still limitations in wound therapy, especially for chronic wounds. Consequently, more effort should be directed towards therapies that restore the regenerative properties of skin (Diegelmann and Evans, 2004). In recent years, polymer materials have been extensively utilized in the preparation of wound dressings for acute wound therapy due to their excellent biocompatibility. These include chitosan, hyaluronic acid, and pectin (Jiang et al., 2016; Graça et al., 2020; Vigani et al., 2019; Amirian et al., 2021; Rezvanian et al., 2017). These polymer-based wound dressings provide a protective barrier and aid in the removal of exudate. However, traditional wound healing dressings offer only a temporary optimal physical environment for the body’s natural healing process, limiting their effectiveness over time. Therefore, fabricating an ideal wound dressing that meets specific requirements for wound care remains a desirable goal.

One effective strategy to enhance the resistance time of wound dressings on damaged skin is to utilize *in situ* gelation. This liquid preparation undergoes a sol-gel transition triggered by factors such as temperature, ion strength, and pH (Soliman et al., 2019). The *in situ* gel offers numerous benefits for drug delivery, including controlled release behavior, precise dosing, and improved patient compliance due to reduced administration frequency. Poloxamers, commonly known as Pluronics^®^, are synthetic copolymers composed of hydrophobic polyoxypropylene oxide (PPO) and hydrophilic polyoxyethylene oxide (PEO) fragments. These polymers are extensively used in the fabrication of thermosensitive hydrogels. At higher temperatures, poloxamers self-assemble into large micelles and crosslink to form hydrogels, exhibiting superior stability against degradation compared to other polymers (Russo and Villa, 2019).

The extract of *Periplaneta americana*, commonly known as the American cockroach, is traditionally used in Chinese medicine to alleviate blood stasis and promote blood circulation. The Kangfuxin (KFX) liquid, which contains PAE and is approved by the China Food and Drug Administration, has demonstrated significant wound healing properties and is widely applied in the treatment of gastric ulcer, ulcerative colitis, and diabetic foot ulcer (Li et al., 2018; Shen et al., 2017). However, despite its longstanding use, the underlying anti-inflammatory mechanism of PAE in excision wounds remains elusive.

In the current study, a thermosensitive *in situ* gel loaded with PAE was developed. Initially, the sol-gel temperature was optimized to enhance the gel’s thermosensitivity. Subsequently, the lyophilized thermosensitive gel’s physicochemical properties, including its thermal behavior, crystallinity, and morphology, were thoroughly characterized. Additionally, the interaction between PAE and F127/F68 was investigated using Fourier Transform Infrared Spectroscopy (FTIR). To assess the gel’s efficacy, both *in vitro* release and *in vivo* wound healing experiments were conducted. Finally, the role of the mitogen-activated protein kinase (MAPK) pathway (p38, ERK, JNK) and inflammatory cytokines (IL-1β, SOD, TNF-α, etc.) in mediating the wound healing effects of PAE was evaluated, providing further insights into its underlying mechanism.

## 2 Materials and methods

### 2.1 Materials

*Periplaneta Americana* extract (PAE) was procured from a Good Agricultural Practice (GAP) certified breeding base located in Sichuan, China. Pluronic P188 (F68, Cat# 50259529) and Pluronic P407 (F127, Cat# 50259528) were supplied by BASF Co., Ltd. in Shanghai, China. Phosphate (Cat# C4135) was obtained from Sigma Chemical Co., Ltd., also based in Shanghai. The ELISA kits for measuring TNF-α (Cat# PT516), IL-1β (Cat# PI303), IL-6 (Cat# PI328), and PGE2 (Cat# PI615) were purchased from Beyotime Biotechnology in Shanghai. Additionally, ELISA kits for SOD (Cat# E-BC-K020-M), GSH (Cat# E-BC-K030-M), MDA (Cat# E-BC-K025-M), and FRAP (Cat# E-BC-K225-M) were acquired from Elabscience Biotechnology Co., Ltd. in Wuhan, China. TRIzol (Cat# 12183555), a cDNA (Cat# 11917020) Synthesis Kit, PVDF membranes (Cat# 88520), a protease inhibitor cocktail (Cat# 78429), phosphatase inhibitors (Cat# 78440), and a BCA kit (Cat# A55864) were sourced from ThermoFisher Scientific in Waltham, United States. The primary antibodies for p-JNK (Cat# ab307802), p-p38 (Cat# ab31828), p-ERK (Cat# ab229912), NF-κB (Cat# ab220803), as well as the secondary antibody (Cat# ab288151, 150113), were provided by abcam in Shanghai. All other chemicals used in this study were of analytical grade.

### 2.2 Preparation of the thermosensitive *in situ* gel

The preparation of the PAE-loaded thermosensitive *in situ* gel involved varying ratios of F127 and F68, as detailed in Table 1. Initially, the appropriate quantities of F127 and F68 were dissolved in distilled water under stirring until a homogeneous, clear solution was achieved. This solution was then stored in a refrigerator overnight. Subsequently, PAE (approximately 1% w/v) was added and dissolved into the polymer solution, ensuring a clear, lump-free solution.

**TABLE 1:** Sol-gel transition temperature of PAE thermosensitive *in situ* gel with varying polymer concentrations.

Formulation	PAE content (%)	Polymer concentration (%, w/v)		Sol-gel transition temperature (°C)
		F127	F68	
F1	1	15	3.75	36.1 ± 0.3
F2	1	17.5	3.75	32.5 ± 0.4
F3	1	20	3.75	27.5 ± 0.6
F4	1	17.5	7.5	36.5 ± 0.8
F5	1	17.5	0	26.3 ± 0.5

### 2.3 Measurement of sol-gel transition temperature by rheometer

The sol-gel transition temperature was accurately determined using an AR 2000ex rheometer equipped with parallel plates, following a previously reported method (Yang et al., 2012). The temperature range was set from 20 °C to 90 °C, incremented in steps of 2 °C, with a fixed gap height of 1.05 mm between the top (35 mm diameter) and bottom stainless-steel plates. An oscillation frequency sweep test was conducted at a controlled strain of γ = 0.01 rad/s, with the frequency varying from 1 to 100 rad/s. The resulting values of the storage modulus (G′) and loss modulus (G″) were plotted against temperature, and the sol-gel transition temperature was identified as the intersection point of G′ and G″.

### 2.4 Differential scanning calorimetry (DSC)

A thorough thermodynamic analysis was conducted on lyophilized PAE hydrogel, pure PAE, F127, and F68 using a Mettler-Toledo differential scanning calorimeter. Samples of approximately 3∼5 mg of powder were precisely weighed and carefully placed in hermetically sealed aluminum pans. A controlled heating rate of 10 °C/min was applied from 30 °C to 200 °C in a nitrogen-rich atmosphere for sample scanning. The melting point of the PAE was accurately determined by identifying the endothermic peak on the DSC curve recorded during this process.

### 2.5 Powder X-ray diffraction (PXRD)

X-ray powder diffraction (XRPD) patterns for various samples were captured utilizing an X-ray diffractometer (Xpert PRO, Panalytical, Germany), employing Cu-Kα radiation at 45 kV and 40 mA. The samples underwent analysis within a 2θ range spanning from 5° to 50°, with a step width of 0.03° and a count time of 2 s per step.

### 2.6 Scanning electron microscopy (SEM)

The morphological characteristics of pure PAE, F127, F68, and lyophilized PAE hydrogel were investigated using a NOVA nano SEM (FEI Company, Hillsboro, Oregon, United States) operated at an accelerating voltage of 3 kV. Prior to imaging, a minute quantity of each sample was evenly spread onto double-sided adhesive tape adhered to an aluminum stub. Subsequently, the mounted samples were coated with platinum using a 208 HR Cressington Sputter Coater (England, United Kingdom) at 40 mA for 120 s. Photographs were captured at varying magnifications to capture the intricate surface features of each sample.

### 2.7 Fourier transforms infrared spectroscopy (FTIR)

To explore potential interactions between PAE and F127 or F68, a comprehensive infrared (IR) spectroscopic analysis was conducted on lyophilized hydrogel, F127, F68, and pure PAE. The Cary 600 series IR spectrophotometer (Agilent Technologies, Santa Clara, California, United States) equipped with an attenuated total reflectance (ATR) sample stage was utilized, operating at a resolution of 4 cm^−1^ and performing 16 scans across the spectral range of 400–4,000 cm^−1^. Prior to sample spectrum recording, background scans were collected to minimize interferences from water and CO_2_ signals, ensuring the accuracy of the analysis.

### 2.8 *In vitro* dissolution

A comparative drug release study was conducted between PAE contained in a thermosensitive *in situ* gel and pure PAE powder. Initially, 5 mL of the PAE thermosensitive *in situ* gel was loaded into 50 mL vials and subjected to mild shaking at 100 rpm and 37.5 °C until the sol-gel transition occurred. Following this, 20 mL of PBS 6.8 was added. For the pure PAE comparison, 50 mg of the coarse drug was dissolved in 20 mL of PBS 6.8 media and shaken at 100 rpm under 37.5 °C. At predetermined time intervals, 5 mL samples were withdrawn and replaced with fresh dissolution medium. To quantify the PAE content in each sample, the amount of tyrosine (Tyr) was determined using HPLC analysis (Agilent 1,100, Shanghai) with a reversed phase C18 alkyl silica gel column (250 mm × 4.6 mm, 5 μm). The mobile phase consisted of a mixture of 0.05 mol/L sodium acetate solution (pH 6.5) and acetonitrile. The gradient elution conditions were set as follows: 0–8 min (A 95%); 8–42 min (A 95%–72%); 42–45 min (A 72%–60%); 45–70 min (A 60%–40%); 70–80 min (A 40%–95%). The HPLC analysis was performed with a flow rate of 1 mL/min, an injection volume of 10 μL, and UV detection at 254 nm (Chen et al., 2019). All samples were analyzed in triplicate to ensure accuracy.

### 2.9 Wound healing effect

Male Sprague-Dawley rats weighing between 180–220 g were individually housed in a temperature-regulated environment (25 °C) under a 12-h light-dark cycle. The animal experiments adhered strictly to the institutional guidelines for animal experimentation and received approval from the Laboratory Animal Care Committee of Shengjing Hospital of China Medical University (Approval number: 2020PS1178K). Prior to experimentation, all rats were anesthetized using isoflurane (5.0% for induction, 2.5% for maintenance). A standardized 6-mm diameter excision wound was created on the shaved and 70% alcohol-disinfected dorsal region of each rat. Subsequently, the rats were randomly assigned to four groups of 24 animals each. The treatment groups included daily applications of saline, F127/F68 hydrogel, 1% (w/v) PAE solution, and PAE-loaded thermosensitive *in situ* gel. The wound areas were photographed on days 0, 6, and 12 to monitor healing progress.

### 2.10 Detection of serum cytokines and endogenous pyrogen

Blood samples were collected on days 0, 6, and 12 via the tail vein puncture method. For plasma separation, the collected blood samples were first allowed to clot at room temperature for 30 min. Subsequently, the samples were centrifuged at 3,500 rpm for 10 min at 4 °C. The serum levels of TNF-α, IL-1β, IL-6, PGE2, SOD, GSH, MDA, and FRAP were then measured using ELISA kits, following the manufacturer’s instructions. Briefly, carefully dispense 50 μL of the sample and 50 μL of the assay buffer into individual wells of a 96-well plate, followed by incubation at room temperature for a duration of 120 min. Subsequently, wash the plate five times using the buffer, and then introduce 100 μL of biotinylated antibody into each well. Seal the reaction wells securely with a plate sealer and maintain incubation at room temperature for 60 min. After that, perform another round of five washes, add 100 μL of HRP-labeled Streptavidin to each well, and incubate in the dark at room temperature for 20 min. Proceed with a further five washes, add 100 μL of chromogenic TMB solution per well, and continue incubation in the dark at room temperature for an additional 20 min. Finally, add 50 μL of stop solution to each well, mix thoroughly. The concentrations of these biomarkers were determined through colorimetric analysis at 450 nm using a microplate spectrophotometer.

### 2.11 Real-time PCR (Rt-PCR)

Following the sacrifice of the rats on Day 0, total RNA was extracted, and reverse transcription was performed to synthesize complementary DNA (cDNA). The expression levels of mRNA were subsequently quantified using real-time PCR (Stratagene Mx3000P, Germany) according to the following protocol: 1) an initial denaturation step at 95 °C for 30 s, followed by 2) 35 cycles consisting of a melting step at 95 °C for 15 s and an annealing/extension step at 60 °C for 60 s. The relative expression of mRNA was calculated using the comparative method. The primer sequences used in this study are listed in Supplementary Table S1.

### 2.12 Western blotting

Protein extraction was carried out by sonicating the sample in RIPA buffer containing a protease inhibitor cocktail and phosphatase inhibitors for 3 min on ice. Subsequently, the protein concentration was measured using a BCA kit. Thirty micrograms of the extracted protein was separated via 10% SDS-PAGE and then transferred onto PVDF membranes. The membranes were blocked with 5% non-fat milk at room temperature for 1 h to prevent non-specific binding. Each membrane was then incubated with the appropriate primary antibody overnight at 4 °C to ensure thorough binding of the antibodies to their target proteins. Following the incubation with the primary antibody, the membranes were thawed and incubated with the secondary antibody for 1 h at room temperature. The secondary antibody was diluted with 5% non-fat milk (1:5,000). Finally, the protein bands were quantified using the ImageJ software for accurate and reproducible analysis. The primary antibodies used in this study include p-JNK, p-p38, p-ERK, and NF-κB, each of which was diluted to a final concentration corresponding to a 1:1,000 dilution ratio.

### 2.13 Statistical analysis

All experimental results are presented as mean values ±standard deviation (SD), derived from a minimum of three measurements (unless otherwise noted). Statistical significance of differences was determined using one-way ANOVA, with a probability level set at 0.05.

## 3 Results and discussion

### 3.1 Impact of polymer concentration on the gelation temperature

In this research, a thermosensitive hydrogel for the delivery of PAE to wounded skin was formulated using a combination of F127 and F68. F127, a triblock copolymer composed of hydrophilic ethylene oxide (PEO) and hydrophobic propylene oxide (PPO) segments, has been extensively utilized in the fabrication of thermosensitive *in situ* gels (Bilensoy et al., 2006; Mohamed et al., 2020). However, the sole use of F127 can present challenges such as a relatively short residence time. To overcome this limitation and enhance the gel properties, F68 was incorporated into the hydrogel formulation along with F127 (Soliman et al., 2019; Kurniawansyah et al., 2020).

**FIGURE 1:**
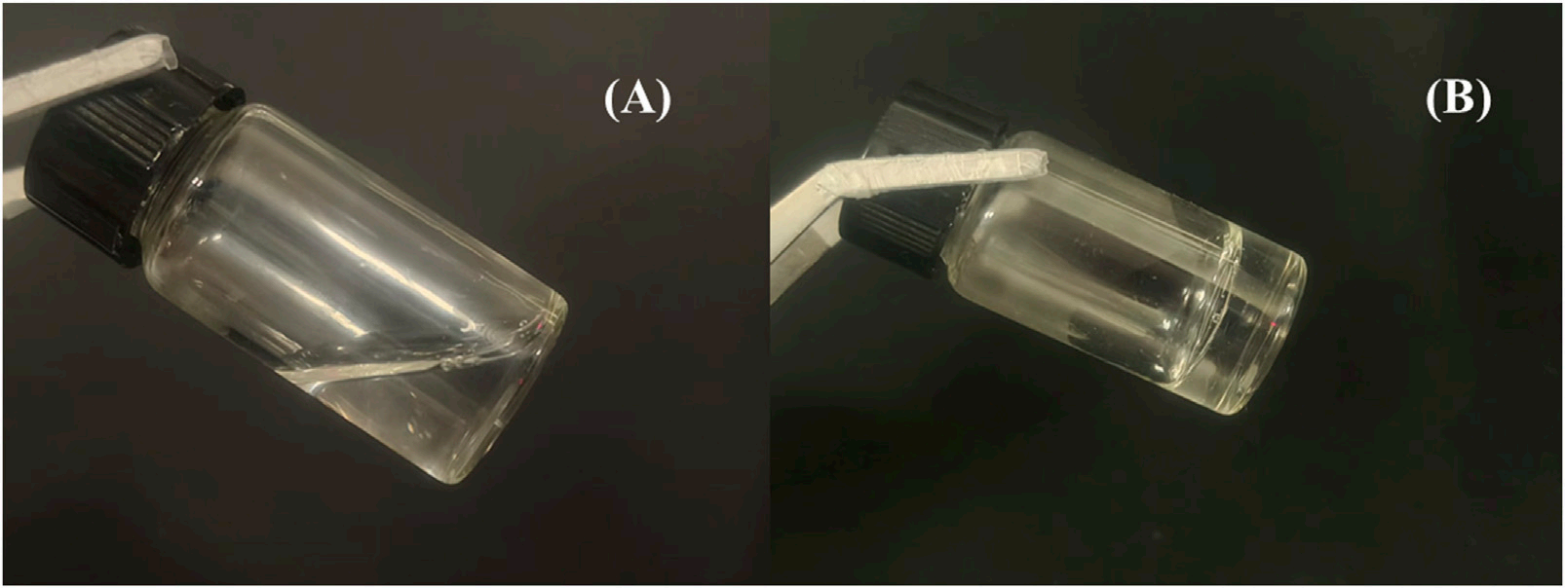
The morphologies of **(A)** F127/F68 thermosensitive *in situ* gel at bellow 32 °C and **(B)** above 32 °C, respectively.

Initially, the sol-gel transition temperatures of the thermosensitive *in situ* gels with varying F127/F68 ratios were evaluated. For effective thermosensitive gel formulations, they should exist in a solution state at room temperature and gel upon contact with skin, typically at a temperature range of approximately 32 °C (Zavgorodnya et al., 2017). As depicted in Table 1, the sol-gel temperature was significantly influenced by the ratio of F127 and F68 (p < 0.05). However, F127 and F68 exhibited differing effects. As the content of F127 increased (F1, F2, and F3), the sol-gel transition temperature decreased. Conversely, an increasing proportion of F68 in the formulation (F2, F3, and F5) led to an elevation in the sol-gel transition temperature, consistent with previous reports (Zavgorodnya et al., 2017; Liu et al., 2018). This disparity can be attributed to the differing PEO/PPO block ratios of F127 and F68. Typically, the hydration of hydrophilic PEO segments and dehydration of hydrophobic PPO segments induce polymer swelling at specific concentrations and temperatures. Since the sol-gel transition temperature is significantly influenced by the PEO/PPO ratios, and a higher proportion of hydrophobic PPO fragments in F127 results in a decreased sol-gel transition temperature, the thermosensitive *in situ* gel system containing 17.5% F127% and 3.75% F68 was selected for further examination (Asasutjarit et al., 2011; Krtalić et al., 2018; Zhang et al., 2017). The morphologies of prepared F127/F68 thermosensitive *in situ* gel bellow and above gelation temperature was exhibited in Figure 1, which presented as solution state at room temperature and become hydrogel at bout 32 °C.

### 3.2 Characterization of the optimized thermosensitive gel

To further elucidate the physiochemical properties of the optimized *in situ* thermosensitive gel, the PAE-loaded thermosensitive gel was lyophilized, and subsequent analyses were conducted to investigate the drug’s existing state and the lyophilized gel’s morphologies.

**FIGURE 2:**
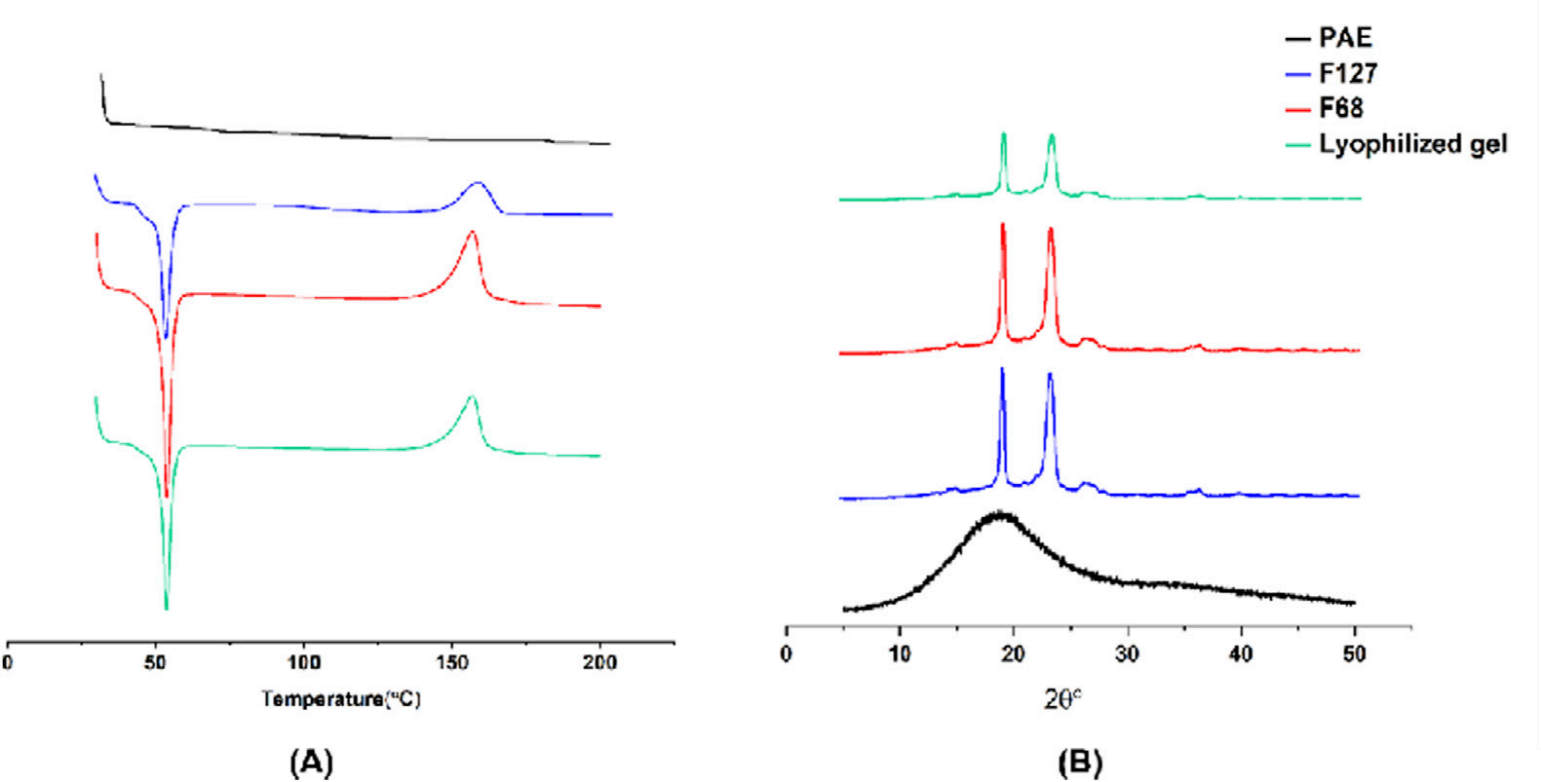
**(A)** DSC curves and **(B)** XRD diffractions of PAE, F127, F68 and the lyophilized thermosensitive gel, respectively.

In this study, the state of PAE within the lyophilized thermosensitive *in situ* gel was characterized using Differential Scanning Calorimetry (DSC) and X-ray Diffraction (XRD). As depicted in Figure 2A, pure PAE exhibited no distinct endothermic peaks, indicating its amorphous nature. Similarly, F127 and F68 displayed similar DSC curves, featuring an endothermic peak at around 53 °C, attributed to their melting point, and an exothermic peak near 155 °C, corresponding to their degradation temperature (Cohn et al., 2006; Dubey et al., 2020). For the lyophilized PAE-loaded thermosensitive gel, a comparable DSC curve was observed, resembling those of F127 or F68, without any additional endothermic or exothermic peaks. This suggests that the PAE retained its amorphous state within the *in situ* thermosensitive gel. This finding was further validated by the XRD study (Figure 2B), where only the characteristic crystalline peaks of F127 and F68 were discernible in the lyophilized gel, confirming the amorphous nature of PAE within the gel matrix (Xiong et al., 2005).

**FIGURE 3:**
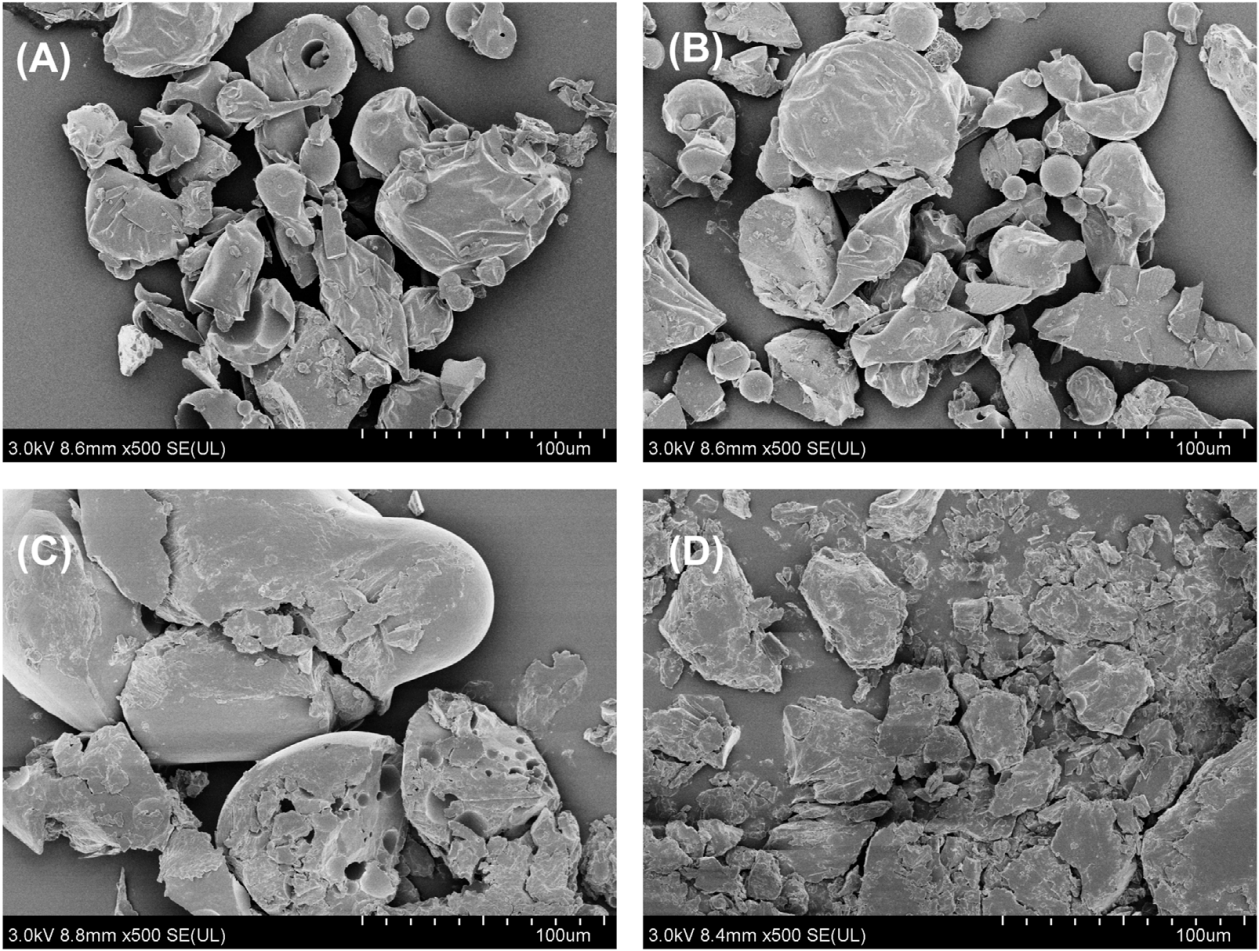
SEM images of **(A)** PAE, **(B)** F127, **(C)** F68, and **(D)** the lyophilized thermosensitive gel, respectively.

Furthermore, the morphological characteristics of pure PAE, F127, F68, and the lyophilized PAE thermosensitive gel were analyzed and are depicted in Figure 3. The pure PAE particles exhibited a bulk and spherical morphology, with a slightly wrinkled surface and a particle size distribution ranging from approximately 20 μm–60 μm. In contrast, F127 and F68 displayed similar spherical shapes but with significantly larger particle sizes. Notably, the lyophilized PAE thermosensitive gel exhibited a relatively smooth surface and bulk morphology, differing from the particulate nature of the pure PAE powder.

### 3.3 Interaction between PAE and F127/F68

**FIGURE 4:**
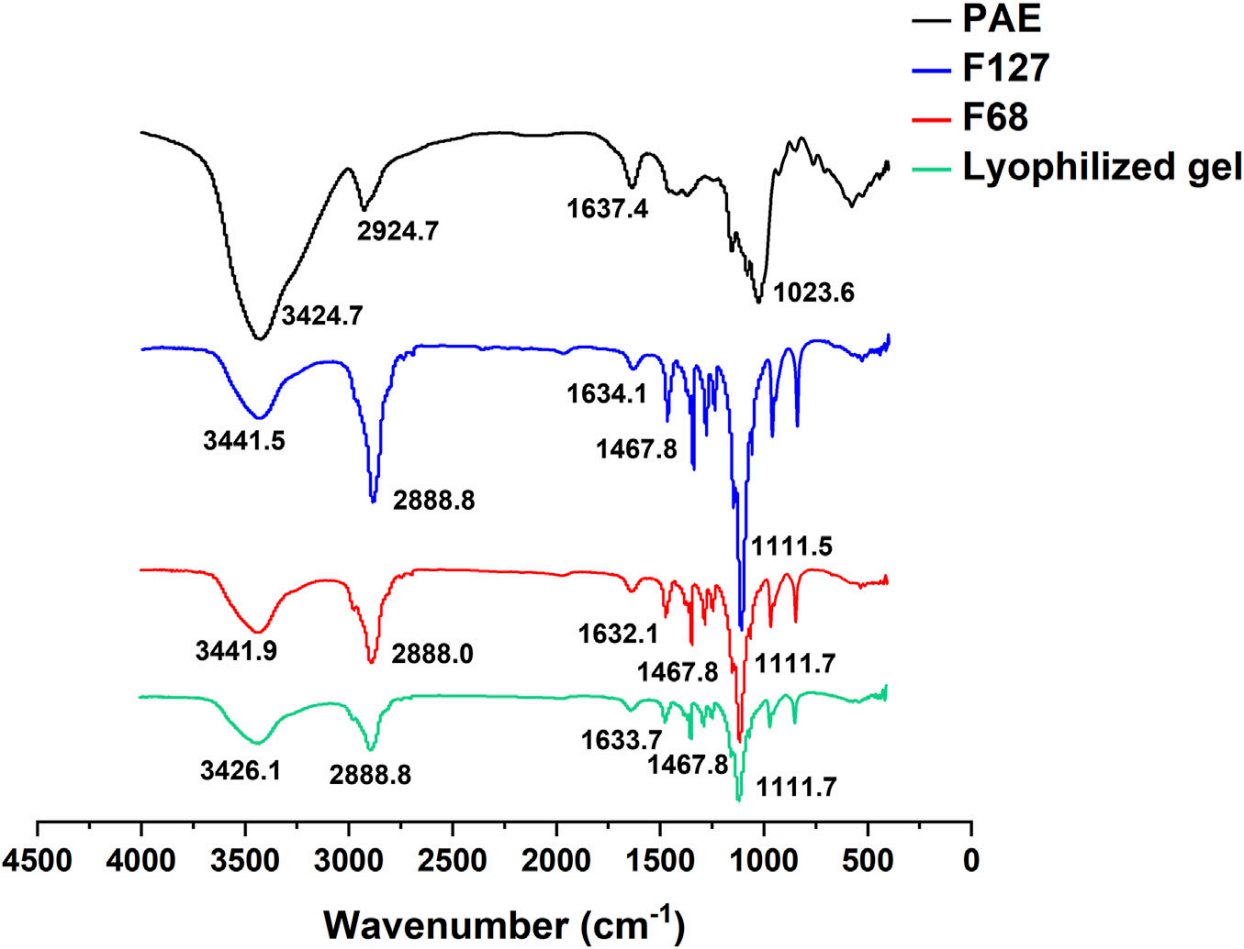
FTIR spectra of PAE, F127, F68, and the lyophilized thermosensitive gel.

To gain insights into the interactions between PAE and F127/F68 within the lyophilized *in situ* thermosensitive gel, Fourier Transform Infrared Spectroscopy (FTIR) analysis was performed on the lyophilized PAE-loaded thermosensitive gel and the individual components. As illustrated in Figure 4, pure PAE exhibited characteristic peaks corresponding to N-H stretching vibration at 3,424.7 cm^-1^, methyl stretching vibration at 2,924.7 cm^-1^, C=N stretching vibration at 1,637.4 cm^-1^, and C-O asymmetric vibration at 1,023.6 cm^-1^ (Chen et al., 2019). For F127 and F68, the O-H stretching vibration peak was observed at approximately 3,441 cm^-1^, along with peaks associated with C-H aliphatic stretching vibration at 2,888 cm^-1^, O-H bending at 1,467 cm^-1^, and C-O stretching vibration at 1,111 cm^-1^ (Agafonov et al., 2021). In the FTIR spectrum of the lyophilized thermosensitive gel, a superposition of the PAE and F127/F68 spectra was observed, accompanied by notable changes. Specifically, the N-H stretching vibration peak of PAE and the O-H stretching vibration peak of F127/F68 shifted to 3,426.1 cm^-1^, suggesting the formation of hydrogen bonds between PAE and F127/F68 within the gel matrix.

### 3.4 Dissolution behavior of PAE thermosensitive hydrogel

**FIGURE 5:**
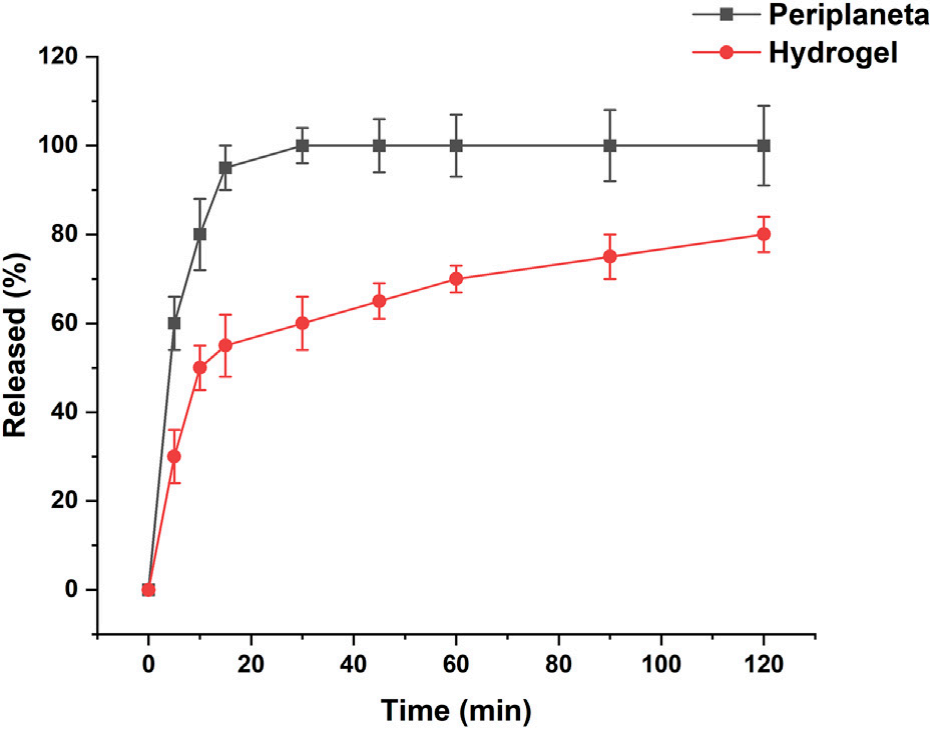
*In vitro* release profiles of pure PAE and PAE-loaded thermosensitive gel in 20 mL of PBS with a pH of 6.8 at 37.5 °C and 100 rpm.

The *in vitro* dissolution kinetics of PAE-loaded thermosensitive hydrogel were evaluated and benchmarked against pure PAE. As depicted in Figure 5, the hydrophilic model drug PAE exhibited a rapid dissolution profile, achieving approximately 100% release within 20 min. In stark contrast, the PAE-loaded thermosensitive gel formulation exhibited a notably slower dissolution rate, particularly during the initial phase (around 30 min), releasing only 60% of the drug within this timeframe. This substantial reduction in dissolution rate can be attributed to the combined effects of the increased viscosity and gel-forming properties of the F127/F68 system. This gel layer formation effectively hinders drug release, thereby promoting a sustained release behavior in the F127/F68 thermosensitive *in situ* gel system (Desai and Blanchard, 1998).

### 3.5 Accelerating wound healing

**FIGURE 6:**
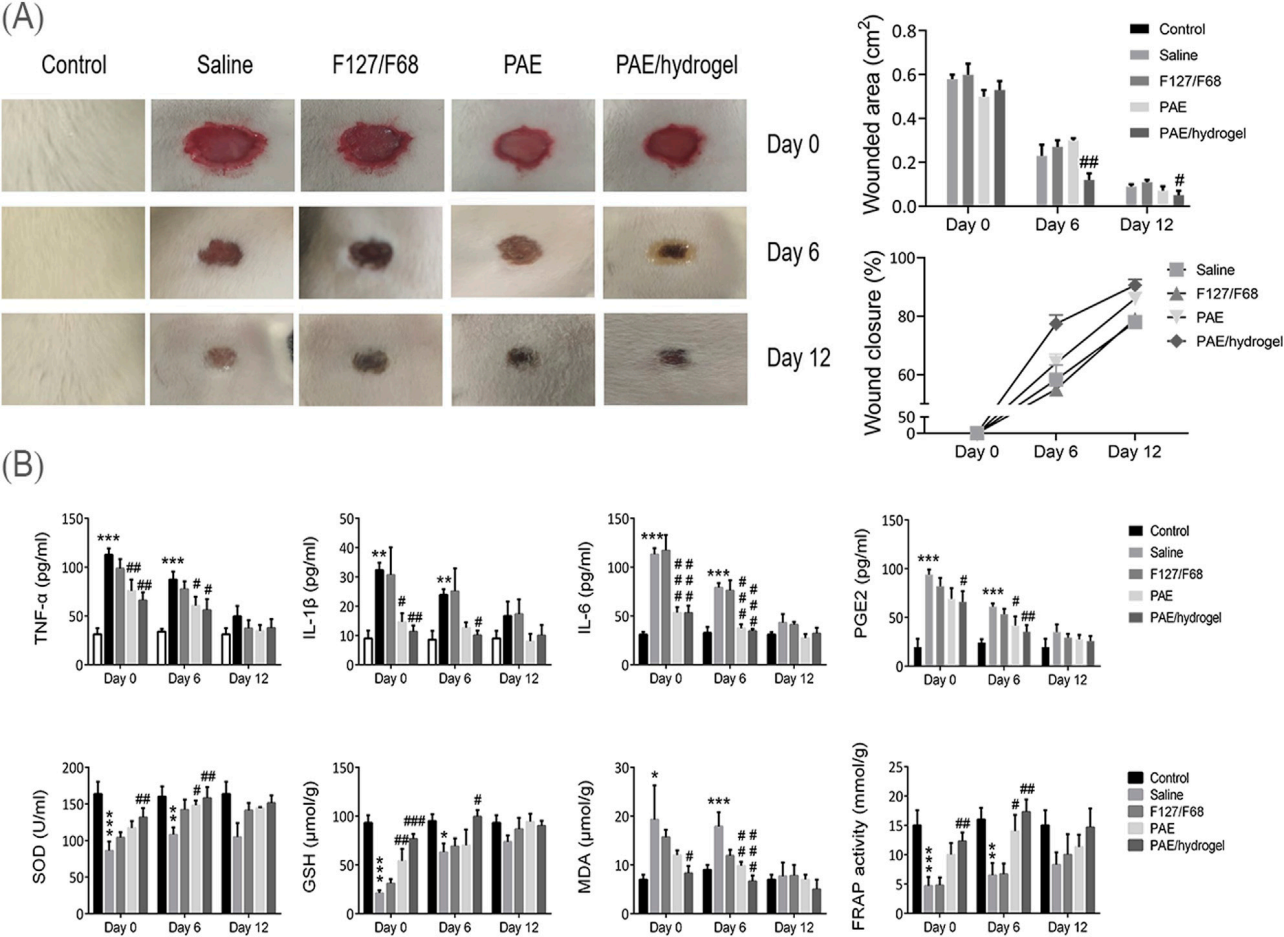
Effects of PAE on pro-inflammatory cytokines and antioxidant activities *in vivo*. **(A)** Photographic representations of the lesion areas, quantification analysis of wound area and closure in rats on Days 0, 6, and 12, demonstrating the progress of wound healing. **(B)** Quantification of serum levels of TNF-α, IL-1β, IL-6, and PGE2 (pro-inflammatory cytokines) as well as SOD, GSH, MDA, and FRAP (antioxidants) in the Control, Saline, F127/F68, pure PAE solution, and PAE/hydrogel groups at 0, 6, and 12 days post-injury using ELISA determination. These data providing insights into the therapeutic efficacy of PAE and its delivery system in modulating inflammatory and antioxidant responses during wound healing.

Previous studies have demonstrated that PAE exhibits beneficial effects on wound healing in clinical and animal models (Zhu et al., 2018). Therefore, the wound healing potential of PAE-loaded thermosensitive *in situ* gel was evaluated using an excision wound model and compared to PAE solution and a blank gel carrier. As shown in Figure 6A, the blank carrier (F127/F68) exhibited no wound healing activity compared to the saline group. Interestingly, both PAE and PAE-loaded thermosensitive gel promoted wound healing from day 6 and 12 post-wounding, with the PAE-loaded gel demonstrating a significantly superior healing effect compared to the PAE solution group. This superiority can be attributed to the advantages of the hydrogel, which provides favorable water vapor permeability and excellent water absorption capacity, thereby maintaining a moist environment around the wound tissue (Xu et al., 2022). Additionally, the prolonged retention time of the hydrogel contributed to its enhanced therapeutic effect (Destruel et al., 2020; Giordani et al., 2019; Gong et al., 2020). The unhealed area of the PAE-loaded thermosensitive gel on days 6 and 12 was significantly smaller than that of the other groups, indicating its superior wound healing effect.

Furthermore, the serum concentrations of pro-inflammatory cytokines such as TNF-α, IL-1β, IL-6, and PGE2, as well as antioxidants including SOD, GSH, MDA, and FRAP, were analyzed and compared in rats. As illustrated in Figure 6B, the PAE-loaded thermosensitive *in situ* gel treated group exhibited the lowest concentrations of TNF-α, IL-1β, IL-6, and PGE2, apart from the control group, on each day of observation. Conversely, the PAE-loaded thermosensitive gel significantly enhanced the production of SOD, GSH, MDA, and FRAP on Days 0 and 6, which plays a crucial role in pain relief and alleviation of inflammation-related conditions. These findings provide an explanation for the favorable wound healing profiles observed with the PAE-loaded thermosensitive *in situ* gel, resulting in nearly complete wound healing after 12 days of treatment.

### 3.6 Mechanism of PAE loaded thermosensitive *in situ* gel on the wound model

**FIGURE 7:**
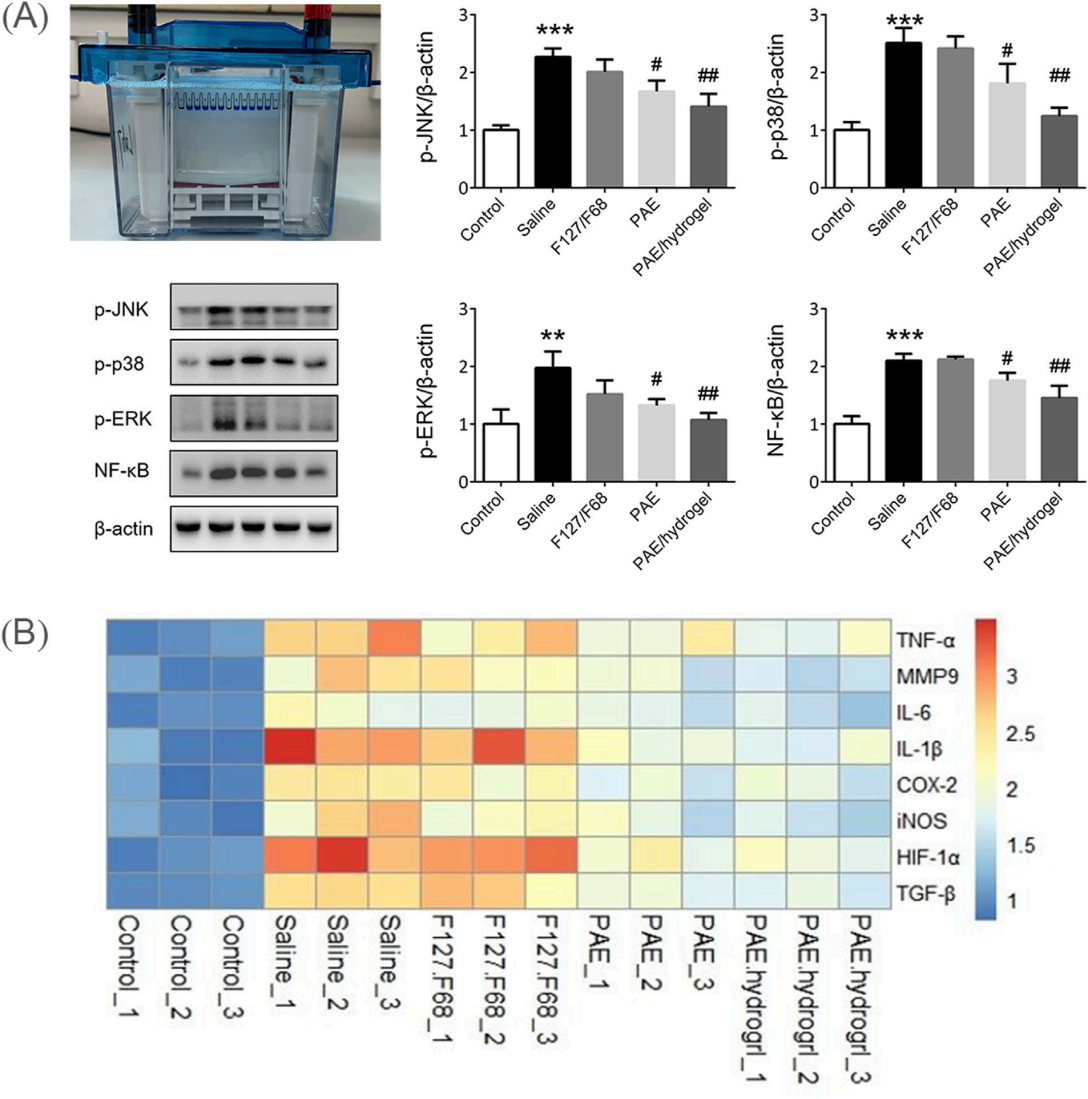
Mechanism of PAE loaded thermosensitive *in situ* gel on the wound model. **(A)** Representative Western blot assays showing p-JNK, p-p38, p-ERK and NF-κB expression (n = 3 per group). **(B)** A heatmap displaying the differential expression of inflammation genes (n = 3 per group). **P < 0.01, ***P < 0.001 vs. Control group; #P < 0.05, ##P < 0.01 vs. Saline group.

To gain further insights into the reduced production of inflammatory factors induced by the administration of PAE thermosensitive *in situ* gel, we investigated the effect of PAE-containing formulations and the control group on the MAPK signaling pathway. The MAPK pathway, including p-JNK, p-p38, and p-ERK, is crucial for regulating the activity and expression of pro-inflammatory cytokines in various cell types (Hunter, 1975; Yang et al., 2022). Activation of this pathway ultimately impacts downstream nuclear transcription factor NF-κB and the production of TNF-α, COX-2, MMP9, and other inflammatory mediators (Li et al., 2021; Ma et al., 2022). Therefore, we measured the expression levels of p-JNK, p-p38, p-ERK, and NF-κB, as well as their transcripts, 24 h after the initial treatment. As depicted in Figure 7A, a significant increase in the expression of p-JNK (P < 0.001), p-p38 (P < 0.001), p-ERK (P < 0.01), and NF-κB (P < 0.001) was observed when the Saline group (p-JNK: 2.27 ± 0.14, p-p38: 2.51 ± 0.26, p-ERK: 1.97 ± 0.29, NF-κB: 2.10 ± 0.12) compared to the Control group (p-JNK: 1.00 ± 0.08, p-p38: 1.00 ± 0.14, p-ERK: 1.00 ± 0.03, NF-κB: 1.00 ± 0.01). Notably, PAE (p-JNK: 1.67 ± 0.19, p-p38: 1.81 ± 0.34, p-ERK: 1.32 ± 0.11, NF-κB: 1.75 ± 0.14) and PAE/hydrogel (p-JNK: 1.41 ± 0.22, p-p38: 1.24 ± 0.15, p-ERK: 1.07 ± 0.12, NF-κB: 1.45 ± 0.21) significantly reduced the expression of p-JNK, p-p38, p-ERK, and NF-κB (PAE: P < 0.05; PAE/hydrogel: P < 0.01) compared to the Saline group. Heatmap analysis (Figure 7B) revealed that PAE significantly reduced the levels of IL-1β, COX-2, HIF-1α, and TGF-β, while PAE/hydrogel significantly decreased the levels of TNF-α, MMP9, IL-6, IL-1β, COX-2, iNOS, HIF-1α, and TGF-β. This suggests that PAE/hydrogel is more effective than PAE alone in inhibiting the transcription of a set of inflammatory genes. The detailed results of the Rt-PCR analysis, including fold change and P values, are presented in Supplementary Table S2. In contrast, F127/F68 did not affect the expression of inflammatory genes (p-JNK: 2.01 ± 0.21, p-p38: 2.41 ± 0.21, p-ERK: 1.52 ± 0.24, NF-κB: 2.12 ± 0.05) compared to the Control group. Collectively, our results indicate that PAE loaded thermosensitive gel exhibits a potential anti-inflammatory effect by accelerating the reduction of endogenous inflammatory mediators and blocking the MAPK pathway. Our study demonstrates that incisional wounds trigger activation of the MARK/NF-κB signaling pathway, resulting in elevated expression of downstream inflammatory mediators, including TNF-α, IL-6, and HIF-1α. PAE facilitates wound healing by inhibiting phosphorylation of the key MARK kinases ERK, JNK, and p38, thereby suppressing NF-κB transcriptional activity and its downstream inflammatory cascade. A prior study provided compelling evidence indicating that the activation of the TGF-β1/Smad2/Smad4 signaling pathway constituted one of the key wound-healing mechanisms of the PAE/Film system. This finding also implied that PAE had the ability to enhance re-epithelialization and collagen production during the wound-healing process (Chen et al., 2019).

Wound healing is a complex process that involves both anti-inflammatory and regenerative mechanisms. Therefore, evaluating tissue regeneration markers holds significant importance. Further studies ought to be designed to thoroughly assess the impact of PAE/gel on these markers, which will enable us gain a more comprehensive understanding of its role in wound healing.

## 4 Conclusion

In this study, we investigated the efficacy of PAE loaded F127/F68 thermosensitive *in situ* gel for wound healing, benchmarking its performance against a PAE solution. The thermosensitive gel carrier, exhibiting a sol-gel transition temperature of 32.5 °C ± 0.4 °C, was formulated using 17.5% F127% and 3.75% F68. The PAE, present in both coarse powder and drug-loaded hydrogel, was found to be in an amorphous state, with hydrogen bonds identified between PAE and F127/F68. This thermosensitive *in situ* gel formulation exhibited a sustained release profile, dissolving approximately 60% of PAE within 30 min. Further analysis of wound healing effects and intramammary cytokines revealed that the PAE loaded thermosensitive *in situ* gel demonstrated superior healing activity compared to other counterparts. In summary, the PAE was incorporated into thermosensitive sol-gel formulation which could keep the PAE state in molecular state in solution before application which could improve the dosage accuracy and spread ability when contact with skin. Also, the thermosensitive gelation properties could provide the sustained release and prolonged skin retention.

## Data Availability

The original contributions presented in the study are included in the article/Supplementary Material, further inquiries can be directed to the corresponding authors.
