# Supplementary material for: Exploring the potential of a thermosensitive in situ gel with Periplaneta americana extracts for efficient wound healing
Source: Front Pharmacol. 2025 Sep 1;16:1672818. doi: 10.3389/fphar.2025.1672818 (PMC12434094; doi:10.3389/fphar.2025.1672818)
Supplement: Supplementary file 1 [file Table1.docx]

Table S1. The primers used in the experiment

| Genes | Forward primers | Reverse primers |
| --- | --- | --- |
| *TNF-α* | 5’-ACACACGAGACGCTGAAGTA-3’ | 5’-GGAACAGTCTGGGAAGCTCT-3’ |
| *MMP9* | 5’-AGGATGGTCTACTGGCACAC-3’ | 5’-GTGCAGGACAAATAGGAGCG-3’ |
| *IL-6* | 5’-CTCATTCTGTCTCGAGCCCA-3’ | 5’-CTGTGAAGTCTCCTCTCCGG-3’ |
| *IL-1β* | 5’-GGGATGATGACGACCTGCTA-3’ | 5’-TGTCGTTGCTTGTCTCTCCT-3’ |
| *COX-2* | 5’-AAAGGCCTCCATTGACCAGA-3’ | 5’-TCGATGTCATGGTAGAGGGC-3’ |
| *iNOS* | 5’-GTTTGACCAGAGGACCCAGA-3’ | 5’-GTGAGCTGGTAGGTTCCTGT-3’ |
| *HIF-1α* | 5’-GCGATGACACGGAAACTGAA-3’ | 5’-TCTTGAATCTGGGGCATGGT-3’ |
| *TGF-β* | 5’-TCGCTTTGTACAACAGCACC-3’ | 5’-ACTGCTTCCCGAATGTCTGA-3’ |
| *Gapdh* | 5’-CAGCAGCCTCGTCTCATAGA-3’ | 5’-TGACTGTGCCGTTGAACTTG-3’ |
