# Supplementary material for: Exploring the potential of a thermosensitive in situ gel with Periplaneta americana extracts for efficient wound healing
Source: Front Pharmacol. 2025 Sep 1;16:1672818. doi: 10.3389/fphar.2025.1672818 (PMC12434094; doi:10.3389/fphar.2025.1672818)
Supplement: Supplementary file 2 [file Table2.doc]

Table S2. PAE or PAE/hydrogel inhibited MAPK-NF-κB signaling pathway

| Gene symbol | Description | Fold change  (Saline *vs* Control) | Fold change  (F127/F68 *vs* Saline) | Fold change  (PAE *vs* Saline) | Fold change  (PAE/hydrogel *vs* Saline) |
| --- | --- | --- | --- | --- | --- |
| *TNF-α* | Tumour Necrosis Factor alpha | 2.78*** | 0.85 | 0.74 | 0.68# |
| *MMP9* | Matrix metallopeptidase 9 | 2.41*** | 0.93 | 0.75 | 0.65# |
| *IL-6* | Interleukin 6 | 2.04*** | 0.93 | 0.84 | 0.75# |
| *IL-1β* | interleukin-1beta | 3.11*** | 0.94 | 0.63## | 0.58## |
| *COX-2* | cyclooxygenase-2 | 2.43*** | 0.91 | 0.72# | 0.74# |
| *iNOS* | Inducible nitric oxide synthase | 2.50*** | 0.84 | 0.72 | 0.64# |
| *HIF-1α* | Hypoxia-inducible factor-1alpha | 3.09*** | 0.98 | 0.67## | 0.62## |
| *TGF-β* | transforming growth factor-beta | 2.57*** | 0.99 | 0.73## | 0.68## |

#Indicates *P* < 0.05 level of significance; ##Indicates *P* < 0.01 level of significance; ***Indicates *P* < 0.001 level of significance
